# Association between dietary intake of protein and amino acids and sarcopenia: a cross-sectional study

**DOI:** 10.1371/journal.pone.0337095

**Published:** 2025-11-21

**Authors:** Weixia Yuan, Panpan Ao, Yun Ma, Ying Ma, Juan Song, Shaofeng Wei, Lijia Yuan

**Affiliations:** 1 School of Public Health, Guizhou Medical University, Guiyang, Guizhou, China; 2 Department of Nutrition, Joint Logistics Support Force 925th Hospital, Guiyang, Guizhou, China; University Hospital of Padova, ITALY

## Abstract

**Objective:**

To research the effects of diet on sarcopenia, and to examine the association of protein and amino acid sources with the risk of sarcopenia in older adults.

**Methods:**

From December 2023 to July 2024, 84 patients with sarcopenia and 173 without sarcopenia were included in the study at the Joint Logistics Support Force 925th Hospital. We compared the in diet and general characteristics between the two groups. Multivariate logistic regression analysis was performed to assess the effects of total protein, animal protein, plant protein, and amino acid on the risk of sarcopenia in older adults.

**Results:**

In terms of nutrient intake, the dietary intakes of energy, protein, fat, carbohydrates, and dietary fiber were significantly lower in the sarcopenia group than the non-sarcopenia group (P < 0.05). Analysis of protein showed that the highest tertile of total protein and animal protein intake was associated with lower incident sarcopenia risk (P < 0.05), while plant protein intake showed no significant association. Further analysis of amino acid showed that the highest tertile of leucine, glutamate, cystine, and tyrosine intake was associated with increased risk of sarcopenia, whereas arginine intake was linked to a lower incident sarcopenia risk (OR: 0.442, 95% CI: 0.200 ~ 0.979; P-trend = 0.038).

**Conclusions:**

The insufficient intake of animal protein and amino acids may be closely associated with the risk of sarcopenia in older adults. Moderately increasing the intake of arginine and animal protein may help reduce the risk of sarcopenia in older adults.

## Introduction

Sarcopenia is an age-related condition characterized by the progressive loss of skeletal muscle mass, strength, and function [1]. According to a report from the United Nations, the older adult population is projected to increase to approximately 2 billion by 2050 [2]. As age increases, the incidence of sarcopenia rises accordingly. Epidemiological data from China indicate that the prevalence of sarcopenia among community-dwelling older adults ranges from 8.9% to 38.8%, reaching up to 67.1% in in individuals aged 80 years and above [3]. Sarcopenia not only increases the risk of falls and fractures in older adults, but is also closely associated with cardiovascular disease, respiratory disorders, and cognitive impairment, contributing to increase the risk of hospitalization, healthcare costs, and mortality risk in older adults [4]. The etiology of sarcopenia in older adults is multifactorial, largely stemming from age-related factors such as a higher incidence of chronic diseases, polypharmacy (use of multiple medications), and psychosocial or behavior factors including depression and sleep disturbances [5]. Furthermore, nutrition, a modifiable lifestyle factor, plays an important role in the onset and progression of sarcopenia [6].

In recent years, dietary factors, particularly protein intake, as a modifiable risk factor has been extensively studied in relation to sarcopenia. Protein is essential for maintaining skeletal muscle growth and are primarily obtained through dietary intake. They help older adults maintain and improve muscle mass and function [7–10], which contributes positively to the prevention and management of sarcopenia. Dietary protein can enhance protein turnover in skeletal muscle tissue, stimulate muscle protein synthesis, and inhibit protein degradation, thus supporting nitrogen balance [11,12]. Research has demonstrated that dysregulation of protein synthesis, degradation, and autophagy plays a key role in the molecular pathogenesis of sarcopenia [13].

However, research has yielded inconclusive results about the relationship between types of proteins and amino acids and muscle loss. In terms of protein quality, animal protein is considered more effective in maintaining muscle health. Previous studies have found that individuals with a high intake of animal protein tend to have greater lean body mass and skeletal muscle quality [8,14]. In the study by Mengjie Yuan et al., higher animal protein intake was found to be associated with lower risks of functional impairment and greater preservation of grip strength in adults over the age of 50 years [15]. In a randomized controlled trial conducted by Robin M Daly, it was found that a diet enriched with lean red meat can significantly improved total body and leg lean tissue mass and leg muscle strength in community-dwelling elderly women [16]. However, Gazzani et al. highlighted the positive effects of plant proteins on somatic function and believe that this may be related to other components in plant-based foods that affect muscle mass and strength [17]. The study by Jacintha Domić et al. observed no significant differences in muscle mass and strength between older men following a lacto-ovo-vegetarian diet and those on a beef-containing diet during a 12-week resistance training program [18]. Therefore, controversy remains regarding the association between proteins from different dietary sources and the risk of sarcopenia. The quality of protein and its amino acid content varies greatly between animal- and plant-based foods, which may explain the heterogeneity between studies. The study by Gorissen et al. found that there are significant differences in the essential amino acid content and amino acid composition between various plant-based protein isolates. Furthermore, compared to animal proteins, plant proteins contain on average 11% less essential amino acids [19]. Amino acids, the basic units that constitute proteins, have recently shown their importance in the field of sarcopenia research [20–24].

Previous studies have shown that changes in the plasma amino acid were associated with low muscle mass among older adults [25]. The metabolism of amino acids, such as aspartic acid and glutamic acid, might play an essential role in regulating muscle mass and strength [26]. However, a study showed that the levels of isoleucine, leucine, tryptophan, serotonin, and methionine in the participants with low muscle quality were significantly higher than that in the participants with high muscle quality, which may be attributed to impaired metabolism of amino acids, resulting in reduced uptake of skeletal muscle, and thus increased circulating plasma amino acid levels [27]. Patricia et al [28] reported that arginine-containing nutritional supplements enhance muscle strength and mass. Similarly, Børsheim et al [29] found that supplementation with essential amino acids (EAAs) plus arginine improved lean body mass, muscle strength, and physical performance in elderly individuals with glucose intolerance. The current evidence on the impact of different types of amino acids on the risk of sarcopenia is inadequate, and there is not a consensus, thus further research is essential.

To better understand strategies for preventing sarcopenia, this study examines the relationship between dietary protein sources, amino acid, and sarcopenia in older adults. The aim is to provide evidence to support the development of dietary guidelines for sarcopenia prevention, thereby contributing to more effective risk reduction in this population.

## Methods

### Study participants

**Fig 1:**
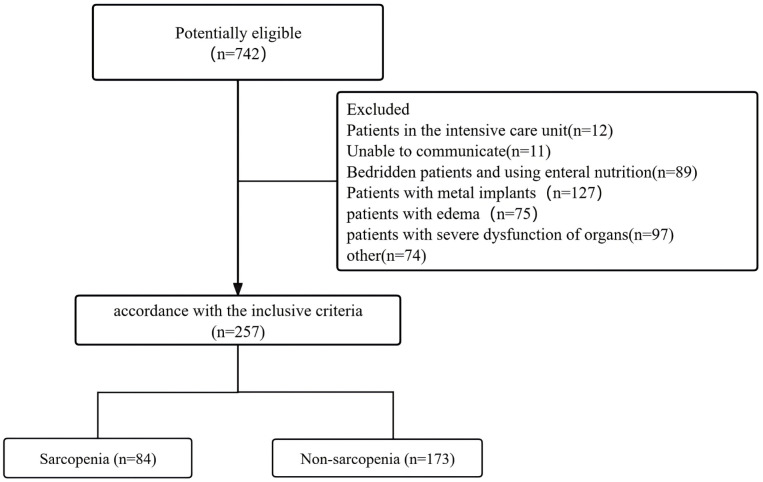
The flowchart of patient selection.

This study included 257 older adult patients, including 84 patients with sarcopenia and 173 without sarcopenia, who were admitted to the Joint Logistics Support Force 925th Hospital from December 2023 to July 2024. The inclusion criteria for this prospective study were as follows: [1] over 60 years old; [2] meet the diagnostic criteria of sarcopenia in the Chinese Expert Consensus on Prevention and Control of sarcopenia in the older adults (2023) [30]. Exclusion criteria: [1] patients with severe cognitive impairment and mental illness unable to take the test; [2] patients with metal implants; [3] patients with severe dysfunction of heart, liver, kidney, and other organs; [4] patients with edema; [5] patients with severe sepsis or systemic inflammatory reaction syndrome; [6] bedridden patients and using enteral nutrition. Fig. 1 shows the flowchart detailing the patient selection process.

This study was conducted according to the guidelines laid down in the Declaration of Helsinki and all procedures involving human participants were approved by the Ethics committee of the Joint Logistics Support Force 925th Hospital; Ethics number: YNKT20230601. The study procedures and protocol were approved and registered with the Chinese Clinical Trial Registry (ChiCTR2300078053). All patients provided written informed consent prior to participation.

### Dietary intake

According to the results of the questionnaire and pre-experiment, the simple food-frequency questionnaire (FFQ 25) was selected for dietary survey [31]. Subsequently, self-reported data of food consumption frequency and food weight estimation were converted into daily intake expressed in grams. Next, using the food classification system of the FFQ 25, we matched these values with the average nutrient content of corresponding food categories from the “China Food Composition Tables Standard Edition” [32] to estimate each individual’s daily total nutrient intake. For each major food category, nutrient composition values were derived by averaging data from several representative foods known to contribute substantially to key nutrients, rather than incorporating all available food items within the category [33–35]. In the statistical analysis of nutrient intakes, total energy intake if it fell below 300 kcal or exceeded 5000 kcal were excluded, as these were considered implausible.

### Sarcopenia

Sarcopenia is defined as low muscle mass combined with either low muscle strength or low physical performance, as stated in the Chinese expert consensus on prevention and intervention for elderly with sarcopenia (2023). Muscle mass was measured using a body composition analyzer (InBody 770). A skeletal muscle index (SMI) of less than 5.7 kg/m² for women or 7.0 kg/m² for men was considered as low muscle mass. The dominant hand grip strength was assessed according to the standardized grip strength measurement guidelines [36]. The thresholds for low muscle strength were set at a hand grip strength of less than 28 kg for men and less than 18 kg for women. Physical performance was evaluated using the 5-time sit-to-stand-test and the 6-meter gait speed test. Low physical performance was defined as a 5-time sit-to-stand-test time ≥ 12 seconds or a 6-meter gait speed < 1.0 m/s.

### Covariate assessments

Trained research staff conducted the face-to-face interviews to collect data. Demographic characteristics (e.g., age, sex, education level), lifestyles (e.g., smoking status, alcohol drinking), chronic disease history, and medication use were collected using a structured questionnaire.

### Statistical analysis

Categorical data were expressed as numbers and a percentage, and the chi-squared or Fisher’s exact test was employed. We used the t-test for data that had a normal distribution and expressed the data as mean ± standard deviation. We used the Mann-Whitney U-test for data that did not have a normal distribution and expressed the data as median (P_25_, P_75_). Multivariable logistic regression analysis model was used to quantify the influencing factors associated with sarcopenia, and the regression model was performed using forward stepwise selection. Dietary protein and amino acid intake were classified into three categories in tertiles and the lowest tertile was taken as reference. Model 1 was the crude model and model 2 was adjusted for the gender, age, educational level, monthly dietary expenses, marital status, and daily step count. Tests for linear trend were performed by treating the median values of dietary protein and amino acid intake in tertiles as continuous values in logistic models. All analyses were performed by SPSS version 27.0. A two-tailed P-value less than 0.05 was considered significant.

## Results

### Participant characteristics

Table 1 shows the baseline characteristics of patients. A total of 257 patients were included in the study, with the incidence of sarcopenia being 32.68%. The baseline characteristics of participants between sarcopenia and non-sarcopenia are shown in Table 1. There were statistically significant differences in marital status, educational level, daily step count, monthly dietary expenses, and exercise habits (P < 0.05).

**Table 1:** Baseline characteristics between sarcopenia and non-sarcopenia.

	Sarcopenia (n = 84)	Non-sarcopenia (n = 173)	t/Z/2	P
Age, year	79.18 ± 7.60	74.98 ± 7.55	3.71	< 0.001
Gender〔n(%)〕			1.67	0.196
Male	36(42.86)	89(51.45)		
Female	48(57.14)	84(48.55)		
Marital status〔n(%)〕			9.75	0.002
Married	62(73.81)	154(89.02)		
Other	22(26.19)	19(10.98)		
Education〔n(%)〕			21.71	< 0.001
No education	23(27.38)	13(7.51)		
Middle or below	42(50.00)	88(50.87)		
Senior or below	19(22.62)	72(41.62)		
Daily step count〔n(%)〕			20.80	< 0.001
> 6000	27(32.14)	108(62.43)		
≤ 6000	57(67.86)	65(37.57)		
Monthly dietary expenses〔n(%)〕			15.03	< 0.001
<¥500	13(15.48)	7(4.05)		
¥500 ~ 1000	45(53.57)	79(45.66)		
> ¥1000	26(30.95)	87(50.29)		
Previous occupational physical labor intensity〔n(%)〕			0.92	0.630
Mild	38(45.24)	84(48.55)		
Moderate	37(44.05)	66(38.15)		
Severe	9(10.71)	23(13.30)		
Exercise habits〔n(%)〕			11.26	0.004
Never	77(91.67)	130(75.14)		
Occasionally	6(7.14)	23(13.30)		
Daily	1(1.19)	20(11.56)		
Grains (g/d)	232.64 ± 99.97	266.21 ± 71.08	−2.23	0.022
Meat (g/d)	56.6 ± 32.65	84.48 ± 48.01	−4.67	< 0.001
Eggs (g/d)	38.57(17.14, 50.00)	50.00(28.57, 50.00)	−2.03	0.042
Milk (g/d)	133.93(6.43, 250.00)	232.14(71.43, 250.00)	−1.54	0.123
Legumes (g/d)	4.38(1.08, 8.48)	6.00(2.00, 14.00)	−1.17	0.241
Aquatic products (g/d)	5.16 ± 8.97	12.96 ± 23.47	−3.32	0.001
Vegetables (g/d)	251.97 ± 123.95	299.67 ± 131.01	−2.45	0.015
Fruits (g/d)	108.75 ± 89.95	152.95 ± 107.28	−2.87	0.005
Nuts (g/d)	4.71 ± 14.23	6.43 ± 13.02	−0.85	0.399

The intake of grains, meat, eggs, aquatic products, fresh vegetables, and fruits was significantly lower in the sarcopenic group than in the non-sarcopenic group (P < 0.05). There was no significant difference in the intake of milk, legumes, and nuts (P > 0.05).

### Nutrient intake in both groups

Table 2 shows that the nutrient intake in the sarcopenia group was significantly lower than in the non-sarcopenia group (P < 0.05), but there was no significant difference in the proportion of the three major nutrients. The dietary intakes of energy, protein, fat, carbohydrates, dietary fiber, and amino acids were significantly lower in the sarcopenia group than in the non-sarcopenia group.

**Table 2:** Comparison of the nutrient intake.

	Sarcopenia (n = 84)	Non-sarcopenia (n = 173)	t/Z	P
Energy(kcal/d)	1 364.52 ± 449.27	1 675.78 ± 369.77	−4.85	< 0.001
Carbohydrate (g/d)	203.14 ± 81.89	237.92 ± 59.03	−3.06	0.003
Fat(g/d)	42.36 ± 14.08	54.88 ± 16.80	−5.18	< 0.001
Protein(g/d)	47.13 ± 17.36	59.39 ± 14.87	−4.89	< 0.001
Energy satisfaction(%)	0.81 ± 0.23	0.90 ± 0.22	−2.63	0.009
Protein(g/kg BW/d)	0.93 ± 0.32	1.08 ± 0.29	−3.20	0.002
Carbohydrate(%)	58.51 ± 8.76	56.90 ± 7.48	1.35	0.180
Fat(%)	29.20 ± 8.72	29.62 ± 6.73	−0.36	0.718
Protein(%)	13.80 ± 2.19	14.28 ± 2.48	−1.33	0.185
Animal protein(g/d)	18.83(12.58,28.01)	27.64(20.79,35.92)	−4.35	< 0.001
Plant protein(g/d)	26.11 ± 9.78	30.60 ± 6.93	−2.63	0.010
Fiber(g/d)	13.26 ± 4.93	15.95 ± 4.22	−3.98	< 0.001
Phenylalanine(mg/d)	2 629.63 ± 977.37	3 247.36 ± 777.74	−4.46	< 0.001
Tryptophan(mg/d)	653.28 ± 239.21	800.84 ± 187.66	−4.37	< 0.001
Leucine(mg/d)	4 068.88 ± 1513.69	5 083.69 ± 1239.84	−4.70	< 0.001
Isoleucine (mg/d)	2 235.42 ± 862.44	2 798.74 ± 697.65	−4.59	< 0.001
Arginine (mg/d)	2 865.93 ± 1124.87	3 678.85 ± 1060.16	−4.96	< 0.001
Glutamic acid(mg/d)	1 1602.38 ± 4208.02	1 4114.06 ± 3209.36	−4.25	< 0.001
Cystine(mg/d)	1 124.68 ± 456.73	1 355.56 ± 315.97	−3.68	< 0.001
Tyrosine(mg/d)	1 842.15 ± 685.87	2 317.36 ± 580.12	−4.81	< 0.001

### Relationship between dietary intake of protein, amino acids and sarcopenia

#### Association between dietary protein and the risk of sarcopenia.

Table 3 shows the association between protein intake and sarcopenia. In the unadjusted logistic regression model, the highest tertile of protein intake was associated with lower risk of incident sarcopenia compared to the lowest tertile. After adjusting for baseline sociodemographic and lifestyle factors, total protein and animal protein intake remained significantly associated with a reduced risk of sarcopenia. The highest tertile of total protein and animal protein intake was associated with lower risk of incident sarcopenia (total protein: OR 0.357, 95% CI: 0.160 ~ 0.789, P-trend < 0.001; animal protein: OR 0.286, 95%CI: 0.093 ~ 0.874, P-trend< 0.001) compared to the lowest tertile. However, no significant association was observed between plant protein and risk of sarcopenia (P > 0.05).

**Table 3:** Association between quantiles of dietary protein intake and the risk of sarcopenia^a^.

	T1	T2	T3	p trend^b^
Total protein				
Model 1^c^	1.00 (Ref)	0.422(0.217 ~ 0.818)	0.245(0.102 ~ 0.585)	< 0.001
Model 2^d^	1.00 (Ref)	0.522(0.244 ~ 1.117)	0.357(0.160 ~ 0.798)	0.011
Animal protein				
Model 1	1.00 (Ref)	0.441(0.193 ~ 1.005)	0.238(0.098 ~ 0.575)	< 0.001
Model 2	1.00 (Ref)	0.652(0.228 ~ 1.864)	0.286(0.093 ~ 0.874)	0.002
Plant protein				
Model 1	1.00 (Ref)	0.320(0.157 ~ 0.655)	0.450(0.221 ~ 0.914)	0.024
Model 2	1.00 (Ref)	0.460(0.203 ~ 1.043)	0.565(0.252 ~ 1.268)	0.187

a: Odds ratio (OR) and corresponding 95% confidence intervals (95% CIs) were presented. b: P for trend was calculated by treating the median values of dietary protein intake in tertiles as continuous values in logistic proportional hazard models. c: Model 1: crude model. d: Model 2: adjusted for gender, age, educational level, monthly dietary expenses, marital status, and daily step count.

### Association between dietary amino acids and the risk of sarcopenia

Table 4 shows the association between dietary amino acids and the risk of developing sarcopenia. The risk of sarcopenia was significantly associated with leucine, arginine, glutamic acid, cystine, and tyrosine, and showed no association with phenylalanine, tryptophan, and isoleucine. Regression analysis of the tertiles of amino acid intake levels indicated that when comparing the highest and lowest amino acid levels, the risk of sarcopenia was positively associated with leucine, glutamic acid, cystine, and tyrosine, and negatively associated with arginine. The highest tertile of arginine intake was associated with a lower risk of incident sarcopenia (OR: 0.442, 95% CI: 0.200 ~ 0.979; P-trend = 0.038) compared to the lowest tertile.

**Table 4:** Association between dietary amino acids and the risk of developing sarcopenia^a^.

	T1	T2	T3	p trend^b^
Phenylalanine				
Model 1^c^	1.00 (Ref)	0.232(0.114 ~ 0.473)	0.278(0.139 ~ 0.559)	< 0.001
Model 2^d^	1.00 (Ref)	0.310(0.137 ~ 0.702)	0.512(0.236 ~ 1.111)	0.077
Tryptophan				
Model 1	1.00 (Ref)	0.327(0.165 ~ 0.650)	0.313(0.155 ~ 0.629)	< 0.001
Model 2	1.00 (Ref)	0.424(0.196 ~ 0.917)	0.605(0.272 ~ 1.346)	0.171
Leucine				
Model 1	1.00 (Ref)	0.274(0.136 ~ 0.551)	0.232(0.114 ~ 0.474)	< 0.001
Model 2	1.00 (Ref)	0.416(0.191 ~ 0.907)	0.435(0.200 ~ 0.945)	0.030
Isoleucine				
Model 1	1.00 (Ref)	0.286(0.144 ~ 0.567)	0.240(0.117 ~ 0.492)	< 0.001
Model 2	1.00 (Ref)	0.391(0.181 ~ 0.844)	0.483(0.218 ~ 1.072)	0.058
Arginine				
Model 1	1.00 (Ref)	0.384(0.184 ~ 0.659)	0.239(0.121 ~ 0.471)	< 0.001
Model 2	1.00 (Ref)	0.472(0.222 ~ 1.007)	0.442(0.200 ~ 0.979)	0.038
Glutamic acid				
Model 1	1.00 (Ref)	0.247(0.121 ~ 0.505)	0.285(0.141 ~ 0.577)	< 0.001
Model 2	1.00 (Ref)	0.377(0.170 ~ 0.838)	0.449(0.204 ~ 0.989)	0.043
Cystine				
Model 1	1.00 (Ref)	0.219(0.108 ~ 0.443)	0.204(0.099 ~ 0.423)	< 0.001
Model 2	1.00 (Ref)	0.334(0.153 ~ 0.727)	0.388(0.174 ~ 0.868)	0.015
Tyrosine				
Model 1	1.00 (Ref)	0.256(0.127 ~ 0.516)	0.226(0.111 ~ 0.458)	< 0.001
Model 2	1.00 (Ref)	0.346(0.158 ~ 0.760)	0.439(0.203 ~ 0.952)	0.032

a: Odds ratio (OR) and corresponding 95% confidence intervals (95% CIs) were presented. b: P for trend was calculated by treating the median values of dietary protein intake in tertiles as continuous values in logistic proportional hazard models. c: Model 1: crude model. d: Model 2: adjusted for gender, age, educational level, monthly dietary expenses, marital status, and daily step count.

## Discussion

Loss of appetite, limited chewing and swallowing function, and reduced mobility due to aging lead to a decrease in dietary intake among older adults. Aging also reduces the sensitivity of skeletal muscle protein synthesis to dietary amino acids. This means older adults require a higher intake of dietary protein and amino acids to achieve the same rate of muscle protein synthesis as younger adults [37]. A substantial body of research indicates that over 50% of older adults in China consume less dietary protein than the average requirement, with the figure rising to more than 70% among those over 80 years of age [38,39]. Currently, dietary-related factors in older adults with sarcopenia have received widespread attention. However, a comprehensive and systematic understanding of the correlation between dietary proteins, amino acids, and sarcopenia is lacking. This study investigated the correlation of dietary protein, amino acids and sarcopenia, making a positive contribution to their rehabilitation and quality of life improvement.

The results of this study showed that the occurrence of sarcopenia was closely related to dietary factors. Compared to non-sarcopenia individuals, a lower intake of meat, eggs, fish, shrimp, and aquaculture products results in insufficient nutrition and energy intake among older adults with sarcopenia. This insufficiency can cause a decline in the mitochondrial energy metabolism of muscle fibers, affecting muscle function and potentially leading to negative nitrogen balance and muscle atrophy [40]. Livestock and poultry meats are rich of protein and trace elements, which can contribute to muscle protein synthesis and promote muscle function [41–43]. Patients with sarcopenia also have a lower intake of grains and fresh vegetables and fruits, which are great sources of dietary fiber. We also found an unfavorable association of low dietary fiber intake with muscle mass maintenance in older adults. It is likely that dietary fiber can change the composition of intestinal flora and reduce systemic inflammation [44,45], reducing the risk of sarcopenia in older adults, which is consistent with the findings of Montiel-Rojas D et al [46]. At the same time, dietary fiber can also stimulate the production of digestive enzymes, promoting the digestion and absorption of protein [47]. This study initially revealed the effects of sources of nutrient intake on sarcopenia and then investigated the effects of the proteins and amino acids in detail, which have a significant impact on sarcopenia.

Further analysis of the effect of dietary protein on sarcopenia has revealed that animal proteins offer a stronger protective effect against sarcopenia than plant proteins. The possible explanation is that animal proteins exhibit stronger muscle synthesis effects compared to plant proteins. Animal proteins usually have a high digestibility and are rich in amino acids such as leucine and arginine [48]. These amino acids are considered to be the major stimulators of muscle protein synthesis [49], potentially alleviating muscle anabolic resistance in sarcopenic patients [50]. Meanwhile, Lim et al [14] also found that animal protein was positively correlated with muscle mass and strength, but not with plant protein. However, Gazzani et al [17]. suggested that plant protein could increase walking distance and improve physical function in middle-aged and older adults. The inconsistency in the role of animal and plant proteins in sarcopenia may be partly attributed to the different varieties of amino acids present in each type of protein.

Meanwhile, our study analyzed the close association between the intake of different amino acids and the risk of sarcopenia. We discovered that leucine, glutamic acid, cystine, and tyrosine were positively associated with the risk of sarcopenia, which is consistent with the findings of Chae M et al [51]. This mechanism may be attributed to the fact that leucine, tyrosine, and phenylalanine can be metabolized into intermediates of the tricarboxylic acid cycle (TCA cycle), which are then converted to glucose through gluconeogenesis [52,53]. Excess glucose may contribute to the synthesis of fatty acids. On the other hand, alanine, isoleucine, and tyrosine can be converted into components of synthetic fatty acids, such as acetyl-Coenzyme A [54]^[45]^. Excessive body fat is associated with mitochondrial dysfunction in the muscle [55], which may lead to reduced muscle protein synthesis [56]. Chae M found that the beneficial effects of dietary branched-chain amino acid intake on adult skeletal muscle mass were greater in the non-obese/ non-abdominal-obese group [51].

Moreover, arginine intake was inversely associated with the risk of sarcopenia. With increasing arginine intake, the risk of sarcopenia significantly decreased, further emphasizing the central role of arginine in the prevention and treatment of sarcopenia. Chao Hua et al also found that serum arginine level was lower in older women with sarcopenia [57]. This suggests that arginine may have a more beneficial effect on the treatment of senile sarcopenia compared to other amino acids. Through cell biology experiments, it was found that arginine supplementation can increase the activity of inducible nitric oxide synthase, accelerating the rate of protein synthesis, and stimulate the phosphorylation of mTOR and p70S6K to promote protein synthesis [58]. These results provide a theoretical basis for future mechanistic studies.

However, dietary assessment in this study was conducted using a simplified food-frequency questionnaire (FFQ 25). Although our analysis identified several significant associations between amino acid intake and sarcopenia risk, estimating specific amino acids using the FFQ 25 is susceptible to measurement error. These errors stem primarily from two sources: [1] relying on participants’ memory and food weight estimation, [2] and the nutrient calculation methodology of the FFQ 25, which relies on average values from foods that are major contributors to key nutrients rather than incorporating all foods within a category. As a result, this approach may not fully capture the intrinsic variations in nutrient composition among individual foods within broader food categories. While operationally practical, this method is prone to misclassification.

Therefore, the findings of this study particularly those concerning the associations between specific amino acids and sarcopenia should be regarded as preliminary and hypothesis-generating rather than conclusive. Future studies using more precise dietary assessment methods, such as three-day 24-hour dietary recalls, are warranted to validate these results.

This methodological limitation is especially important when interpreting counterintuitive findings, such as the observed positive association between higher leucine intake and increased sarcopenia risk. Given that leucine is widely recognized for its role in promoting muscle protein synthesis, this apparent positive association may reflect residual confounding. One plausible explanation is that individuals in early, undiagnosed stages of sarcopenia may have modified their dietary intake, and the limited precision of the abbreviated FFQ may have inaccurately captured these changes, resulting in a spurious association. Furthermore, this study is a cross-sectional design, and the causality could not be determined. Future studies should to be conducted in a wider population and the findings and related mechanisms of this study need be further verified through cohort studies and animal experiments.

## Conclusions

In conclusion, our findings suggest that lower dietary intake of protein and amino acids may be associated with an elevated risk of sarcopenia. Among older adults with sarcopenia, higher intakes of animal protein and arginine appeared to correlate with a reduced risk of the condition. These results imply that, for older individuals with or at risk of sarcopenia, consuming high-quality animal protein and arginine might be beneficial for maintaining muscle mass and supporting muscle recovery. However, these observational associations should be interpreted with caution, and further research is necessary to validate these relationships and clarify their clinical relevance.
